# Prevalence and Phylogenetic Analysis of Human Bocaviruses 1-4 in Pediatric Patients with Various Infectious Diseases

**DOI:** 10.1371/journal.pone.0160603

**Published:** 2016-08-04

**Authors:** Min Zhao, Runan Zhu, Yuan Qian, Jie Deng, Fang Wang, Yu Sun, Huijin Dong, Liying Liu, Liping Jia, Linqing Zhao

**Affiliations:** Key Beijing Laboratory of Viral Disease Etiology, Laboratory of Virology, Capital Institute of Pediatrics, Beijing, China; Kliniken der Stadt Köln gGmbH, GERMANY

## Abstract

**Objectives:**

Viral infections caused by human bocaviruses 1–4 (HBoV1-4) are more complicated than previously believed. A retrospective, large-scale study was undertaken to explore the prevalence of HBoV1-4 in pediatric patients with various infectious diseases and delineate their phylogenetic characteristics.

**Methods:**

Clinical samples from four specimen types, including 4,941 respiratory, 2,239 cerebrospinal fluid (CSF), 2,619 serum, and 1,121 fecal specimens, collected from pediatric patients with various infectious diseases were screened for HBoV1-4. A 690-nt fragment in each specimen was then amplified and sequenced for phylogenetic analysis. Clinical characteristics of HBoV-positive patients with different specimen types available were evaluated.

**Results:**

Approximately 1.2% of patients were confirmed as HBoV-positive, with the highest positive rate in patients with gastrointestinal infection (2.2%), followed by respiratory (1.65%), central nervous system (0.8%), and hematological infections (0.2%). A single genetic lineage of HBoV1 circulated among children over the 8-year period, while a new cluster of HBoV2, via intra-genotype recombination between HBoV2A and HBoV2B, was prevalent. Some patients had HBoV1-positive respiratory and serum specimens or fecal specimens. Several cases became HBoV1-positive following the appearance of respiratory infection, while several cases were positive for HBoV2 only in CSF and serum specimens, rather than respiratory specimens.

**Conclusions:**

A single genetic lineage of HBoV1 is speculated as a viral pathogen of respiratory infection and causes both comorbid infection and acute gastroenteritis. Additionally, a new cluster of HBoV2 is prevalent in China, which may infect the host through sites other than the respiratory tract.

## Introduction

Since its discovery in nasopharyngeal secretions of children with respiratory tract infections in 2005 by Allander et al. [1], human bocavirus (HBoV) has been suggested as a viral pathogen associated with respiratory infections with incidences ranging from 0.8% to 33% in children. HBoV infection is frequently observed in children with pneumonia, acute wheezing, asthma, or bronchiolitis [2–8], and sometimes in life-threatening lower respiratory infections [9–10].

In 2009–2010, three additional HBoV genotypes (HBoV2-4), which typically occur in the gastrointestinal tract but rarely in the respiratory tract, were reported [11–13]. Additionally, a fatal subacute myocarditis case in a 13-month-old child was recently associated with HBoV2 by Brebion et al. in France [14].

HBoV (family *Parvoviridae*, subfamily *Parvovirinae*, genus *Bocaparvovirus*) is a small, non-enveloped virus. It contains a single-stranded DNA of about 5.3 kb with three open reading frames, which encode two non-structural proteins, NS1 and NP1, and two capsid proteins, VP1 and VP2 [15].

Although HBoV1-4 have been identified globally, predominantly in nasopharyngeal, fecal, cerebrospinal fluid, and blood specimens [2–8], the significance of HBoVs in infectious disease remains unclear [16]. Schildgen et al. discussed that viral infections in general and those caused by HBoVs in particular are more complicated than previously believed, and suggested that the interpretation of molecular findings requires more sophisticated algorithms and a deeper knowledge of the molecular biology of contributing pathogens [17]. To explore the prevalence and phylogenetic characteristics of HBoV1-4 in pediatric patients with infectious disease belonging to different tissues, a retrospective, large-scale study (over an 8-year period) was undertaken to provide an explanation of HBoV molecular biology based on clinical information.

## Materials and Methods

### Study subjects

In the retrospective study, pediatric patients who hospitalized (inpatients) in or just visited (outpatients) the affiliated children’s hospital with infectious disease and specimens collected for routine laboratory testing were included. These patients were categorized into four cohorts according to disease.

For the acute gastrointestinal infection group, the inclusion criterion was patients who experienced three or more loose or liquid stools per day for less than 2 weeks, and the exclusion criterion was patients diagnosed with chronic diarrhea over 2 weeks. Between October 2010 and December 2012, 1,121 fecal specimens were collected from outpatients with acute diarrhea (715 males and 406 females). All fecal specimens were diluted (1:10) in phosphate buffer, vortexed, and centrifuged at 1,500 × g for 15 min. The supernatants were collected in sterile tubes and stored at −20°C before use.

For the acute respiratory tract infection group, the inclusion criterion was patients diagnosed with acute respiratory tract infections, including rhinitis, laryngitis, tonsillitis, tracheitis, bronchitis, bronchiolitis, and pneumonia. From January 2006 to December 2013, 4,941 respiratory specimens (2,900 nasopharyngeal aspirates [NPA] and 2,014 throat swabs [TS]) were collected from inpatients or outpatients (2,892 males and 2,049 females). NPA and TS specimens were processed as previously reported [18].

For the central nervous system infection group, the inclusion criterion was patients diagnosed with viral meningitis or encephalitis. From March 2006 to December 2013, 2,239 cerebrospinal fluid (CSF) specimens were collected from inpatients (1,341 males and 898 females). All CSF specimens were collected in sterile tubes and stored at −20°C until further use.

For the hematologic system infection group, the inclusion criteria were pediatric patients diagnosed with infectious mononucleosis, anemia, idiopathic thrombocytopenic purpura, aplastic anemia, leukemia, or blood cell abnormalities. From January 2006 to December 2013, 2,619 serum specimens were collected from inpatients (1,548 males and 1,071 females). All serum specimens were collected in sterile tubes and stored at −20°C until further use.

The study was approved by the Ethics Committee of the Capital Institute of Pediatrics. Informed consent was waived for participants because remnant NPA specimens were used for HBoV screening.

### Nucleic acid extraction

DNA was extracted from specimens using DNAzol® BD (Molecular Research Center, Cincinnati, OH, USA) and resuspended in 30 μL of 8 mM NaOH.

### Detection of HBoV1-4 by PCR

For HBoV screening, published primers HBoV2-sf2 (5′-TGCTTCAACAGGCAAAACAA-3′) and HBoV2-sr2 (5′-TCCAAGAGGAAATGAGTTTGG-3′) were used to amplify a 495-nt fragment within the open reading frame of NS1 of HBoV1-4 from clinical specimens. The amplification profile comprised an initial denaturation at 94°C for 5 min, followed by 45 amplification cycles (94°C for 30 s, 58°C for 30 s, and 72°C for 45 s). The amplification products were analyzed by electrophoresis on a 1.5% (w/v) agarose gel [12]. For genotype identification by sequencing, a 690-nt fragment at the NP1 and VP1 gene boundary of HBoV-positive samples was amplified using primers HBoV-c1 (5′-CTTYGAAGAYCTCAGACC-3′) and HBoV-c2 (5′-TKGAKCCAATAATKCCAC-3′) designed according to GenBank sequences of HBoV1-4. The amplification profile comprised an initial denaturation at 94°C for 5 min, followed by 45 amplification cycles (94°C for 30 s, 48°C for 30 s, and 72°C for 1 min), with a final 10-min extension at 72°C [18]. PCR products were analyzed by electrophoresis on a 1.5% (w/v) agarose gel. Positive PCR products amplified by primers HBoV-c1 and HBoV-c2 were then sequenced, and phylogenetic analysis was conducted.

To avoid PCR contamination, all procedures including specimen processing, DNA extraction, PCR, and gel electrophoresis were performed in separate areas of the laboratory [19].

### Phylogenetic relationship analysis

A phylogenetic analysis was conducted using the MEGA version 6.0 software package. Phylogenetic trees were constructed using the neighbor-joining method and maximum composite likelihood model using sequences of the target gene from HBoV-positive specimens in the study and HBoV sequences from GenBank. A discrete gamma distribution, used to model evolutionary rate differences among sites (1 category, +G), was constructed in MEGA 6.0. Bootstrap pre-sampling (1,000 replications) was used to assess the reliability of individual nodes in each phylogenetic tree.

### Clinical characteristic analysis of inpatients with different specimen types available

The medical records of HBoV-positive inpatients were reviewed. All specimens from patients with different specimen types available were screened for HBoV genotype. Their profiles and HBoV genotypes are described.

### Statistical analysis

The profiles of HBoV-positive patients according to age were described using the mean ± standard deviation. Pearson’s chi-squared test was used to evaluate differences in HBoV positivity rates between male and female patients, and *p* < 0.05 was considered to be statistically significant.

## Results

### HBoV screening by PCR

Among 4,941 NPA/TS specimens, 82 (1.65%, 82/4.941) were identified as HBoV-positive by PCR. In 1,121 fecal specimens, 25 (2.2%, 25/1,121) were positive; in 2,239 CSF specimens, 18 (0.8%, 18/2,239) were positive; and in 2,619 serum specimens, 6 (0.2%, 6/2,619) were positive. The overall HBoV-positivity rate in all infectious diseases evaluated was 1.2% (131/10,920).

### Phylogenetic analysis of HBoVs

**Fig 1:**
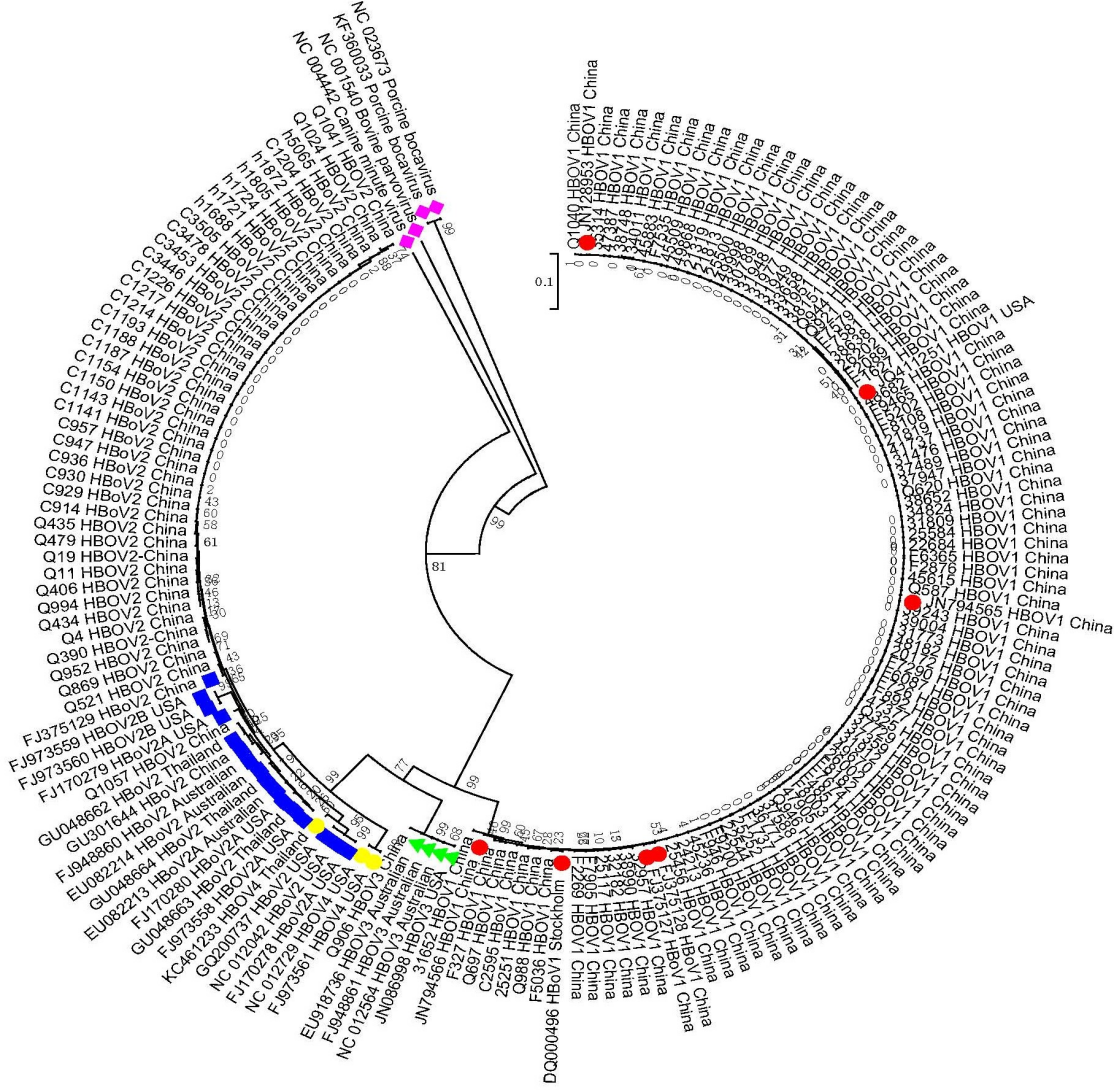
NP1/VP1 gene sequences (n = 131) obtained from clinical samples and GenBank sequences for HBoV1-4 were used for tree construction. The phylogenetic tree was constructed using the neighbor-joining method based on the Kimura -parameter model with 1,000 bootstrap replicates, using the MEGA 6.0 software program. A red dot denotes an HBoV1 strain downloaded from GenBank, a blue box denotes an HBoV2 sequence from GenBank, a green triangle denotes an HBoV3 sequence from GenBank, a yellow diamond denotes an HBOV4 sequence from GenBank, and a purple diamond denotes other parvoviruses. All remaining accession numbers represent the 131 sequences obtained from clinical samples in the present study.

All 131 amplicons of the NP1/VP1 boundary region from HBoV-positive specimens were sequenced and blasted with reference sequences of HBoV1, HBoV2 (2A and 2B), HBoV3, HBoV4, porcine parvovirus, bovine parvovirus, and canine parvovirus from GenBank. The results shown in Fig 1 indicate that these 131 sequences clustered into three groups. The first group, including 82 sequences from NPA/TS specimens, 9 from fecal specimens, and 1 from CSF specimens, shared 97.1–100% identity with HBoV1 reference sequences and was grouped into HBoV1. The second group, including 15 sequences from fecal specimens, 17 from CSF specimens, and 6 from serum specimens, shared 92.4–100% identity with HBoV2 reference sequences and was grouped into HBoV2. The third group, including only one sequence from fecal specimens, shared 99% identity with HBoV3 reference sequences and was grouped into HBoV3. No HBoV4 sequence was detected in this study.

Therefore, phylogenetic analysis indicated that all 82 NPA/TS specimens were identified as HBoV1 (1.65%, 82/4,941). The 25 fecal specimens included 9 (0.80%, 9/1,121) HBoV1, 15 (1.33%, 15/1,121) HBoV2, and 1 (0.08%, 1/1,121) HBoV3 isolate. The 18 CSF specimens included 1 (0.04%, 1/2,239) HBoV1 and 17 (0.75%, 17/2,239) HBoV2 isolates. All six serum specimens (0.2%, 6/2,619) were HBoV2-positive.

**Fig 2:**
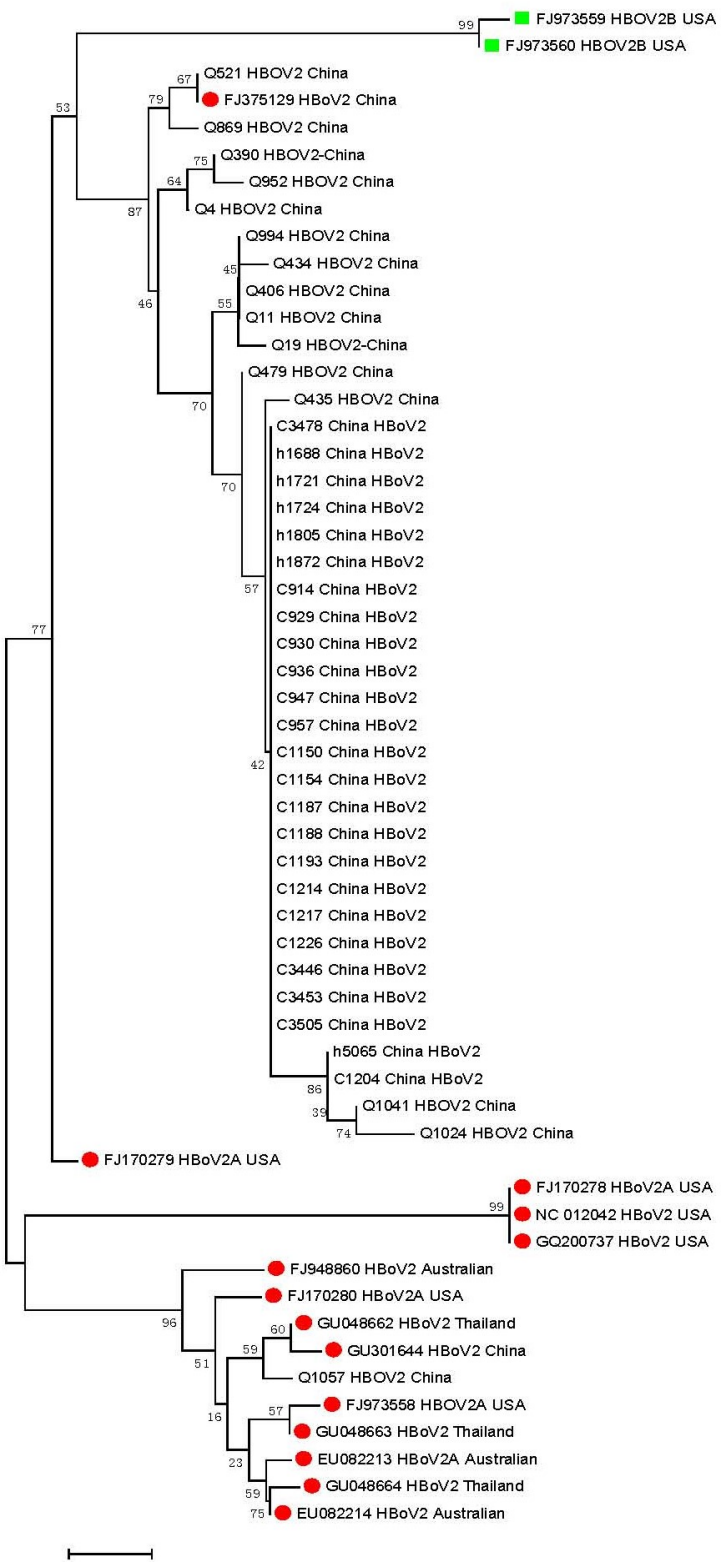
HBoV2 NP1/VP1 gene sequences (n = 38) obtained from clinical samples and GenBank sequences for HBoV2 (n = 16) were used for tree construction. The phylogenetic tree was constructed using the neighbor-joining method based on the Kimura 2-parameter model with 1,000 bootstrap replicates, using the MEGA 6.0 software program. A red dot designates an HBoV2A strain and a green box designates an HBoV2B sequence from GenBank.

Additional phylogenetic analyses were subsequently performed on the 38 HBoV2 sequences (Fig 2). These 38 sequences clustered into two distinct genetic lineages; 1 sequence (Q1057) clustered together with GU048662 from Thailand (99.6% identity) and GU301644 from China (99.4%), while the other 37 sequences clustered with FJ375129 from Shanghai (96.2–99.2% identity).

### Prevalence characteristics of different HBoV genotypes

**Fig 3:**
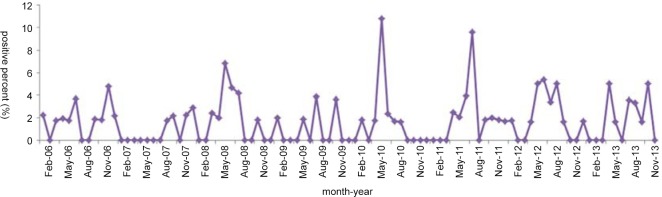
Monthly distribution of HBoV1-positive respiratory specimens from 2006 to 2013.

Of 82 HBoV1-positive patients with respiratory tract infections, 55 were male (1.90%, 55/2,892) and 27 were female (1.31%, 27/2,049). No significant difference in HBoV1-positive rates was noted between male and female patients (χ^2^ = 2.11, *p* > 0.05). The mean age was 1.8±2.15 years (range: 7 days–14 years), with 80.4% (66/82) of patients younger than 2 years old. Among these 82 patients, complete medical records were available for 49, including 22 (44.9%, 22/49) patients co-infected with other pathogens, such as *Mycoplasma*, respiratory syncytial virus, or Epstein–Barr virus, and 27 (55.1%, 27/49) patients solely infected with HBoV1. Analysis of the monthly distribution of HBoV1-positive respiratory samples from 2006 to 2013 revealed peaks in June 2006, December 2007, June 2008, August 2009, June 2010, August 2011, July 2012, and August 2013 (Fig 3).

**Fig 4:**
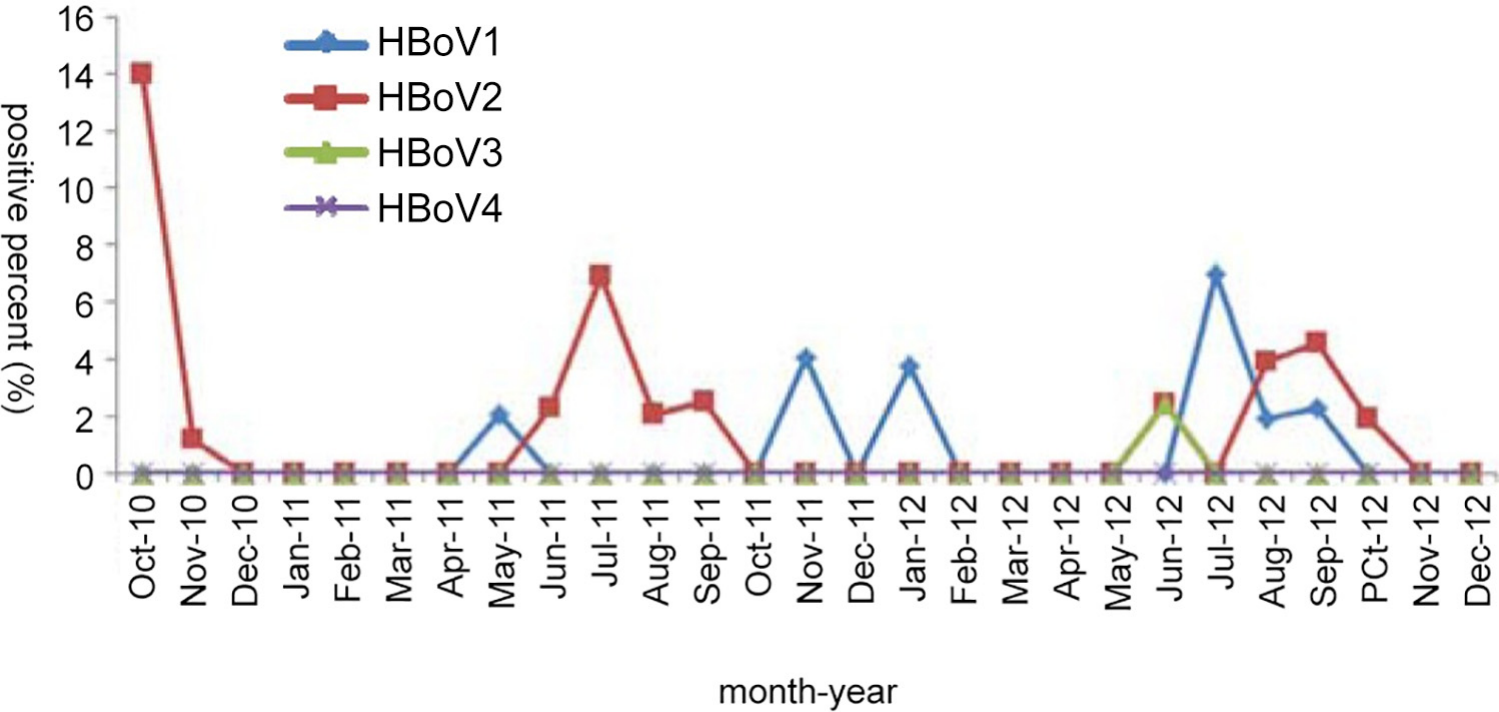
Monthly distribution of HBoV-positive fecal specimens from 2010 to 2012

Of 25 HBoV-positive fecal specimens (2.2%, 25/1,121), the monthly distribution revealed that HBoV1 peaked in November 2011 and July 2012, while HBoV2 peaked in October 2010, July 2011, and September 2012 (Fig 4). All HBoV-positive patients, including 12 males (1.68%, 12/715) and 13 females (3.20%, 13/406), were younger than 2 years old, with a mean age of 0.5±0.39 years (range: 2 months–1.58 years). Additionally, no significant difference in the positivity rates was noted between male and female patients.

**Fig 5:**
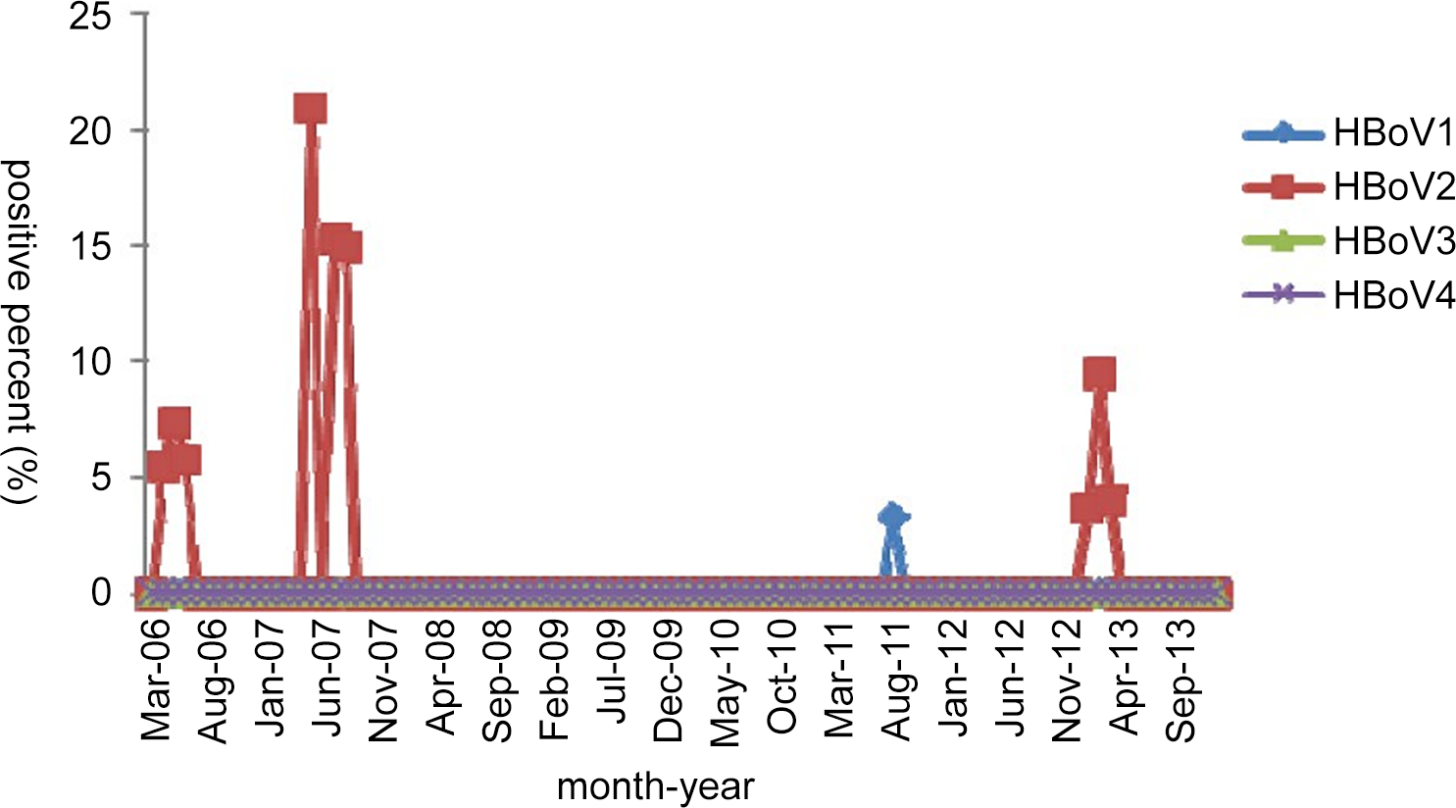
Monthly distribution of HBoV-positive CSF specimens from 2006 to 2013.

For 18 HBoV-positive CSF specimens (0.8%, 18/2,239), the monthly distribution revealed that the 1 HBoV1-positive specimen was found in 2011, and a high HBoV2-positive rate was observed in 2007 (Fig 5). These 18 patients, including 9 males (0.7%, 9/1,341) and 9 females (1.0%, 9/898), showed no obvious age distribution (range: 21 days–14 years). Additionally, no significant difference in the positivity rates was noted between male and female patients.

**Fig 6:**
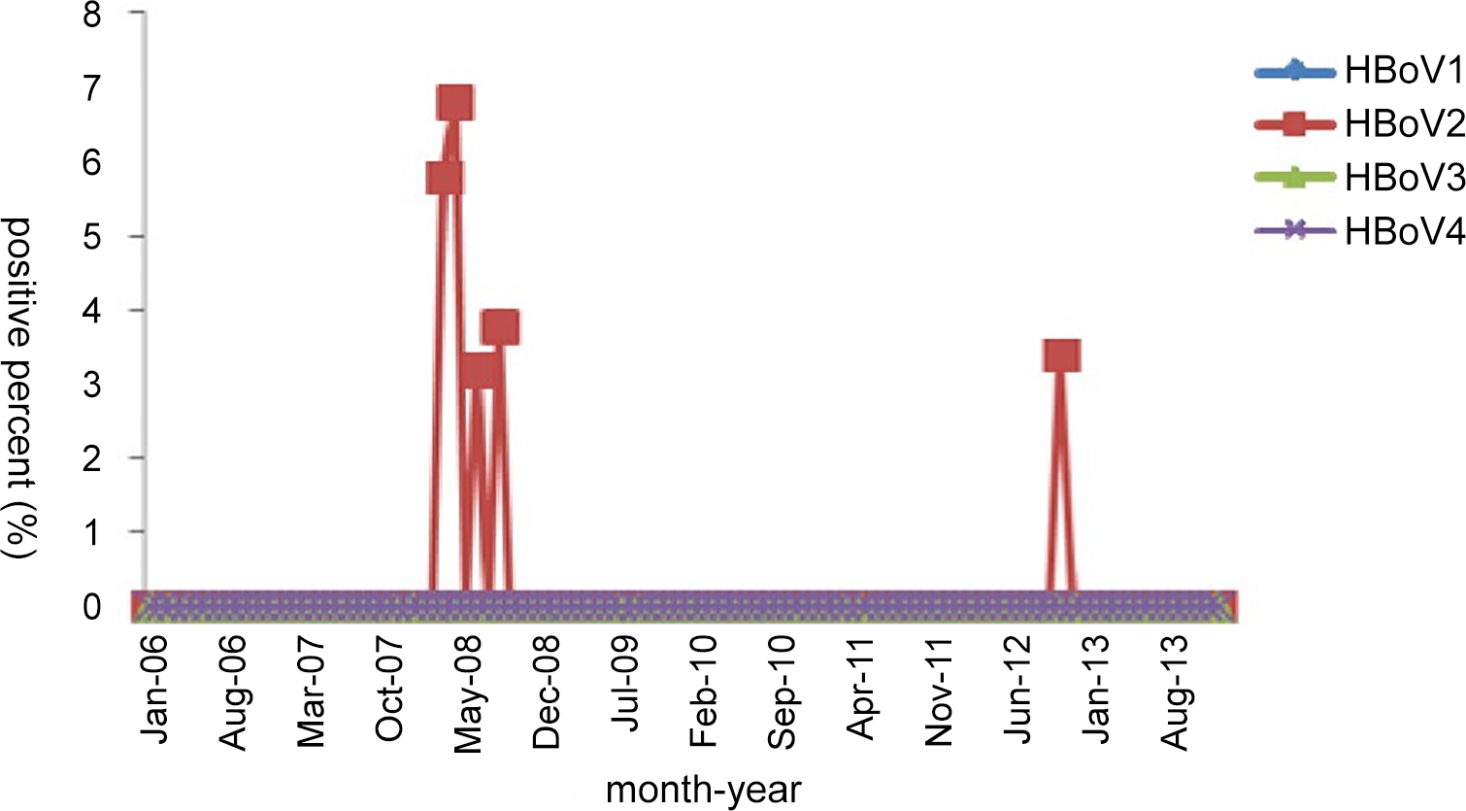
Monthly distribution of HBoV-positive serum specimens from 2006 to 2013.

For the six HBoV2-positive serum specimens (0.20%, 6/2,619), the monthly distribution revealed that a high HBoV2-positive rate was observed in 2008 (Fig 6). These six HBoV2-positive patients, including 5 males (0.32%, 5/1548) and 1 female (0.09%, 1/1071), showed no obvious age distribution (range: 2 months–14 years). Additionally, no significant difference in the positivity rates was noted between male and female patients.

### Clinical characteristics of inpatients with different specimen types available

All specimens from patients with different specimen types available were screened for HBoV genotype. Their profiles and HBoV genotypes are described in Table 1.

**Table 1:** HBoV results of HBoV-positive inpatients with different types of specimens collected for routine laboratory testing.

Case	Sex	Age	Admission date	Major diagnosis	Respiratory specimens			Serum specimens			Fecal specimens			CSF* specimens		
					No.	Date of specimen	HBoVs*	No.	Date of specimen	HBoVs*	No.	Date of specimen	HBoVs*	No.	Date of specimen	HBoVs*
1	m	9.7m	11/22/2013	severe Pn*, acute gastroenteritis	49868	11/25/2013	HBoV1	CMV14456	11/25/2013	HBoV1	CR9245 (RV*, negative)	11/26/2013	HBoV1	C4021	11/25/2013	Negative
2	f	1.5m	9/15/2013	Pn*, purulent meningitis	48182	9/17/2013	HBoV1	CMV13190	9/16/2013	HBoV1				C3855	9/15/2013	Negative
3	m	5m	5/12/2011	severe Pn*, acute gastroenteritis	30789	5/12/2011	HBoV1	h3395	5/13/2011	Negative	CR7110 (RV*, positive)	5/16/2011	HBoV1			
4	f	49.2m	1/16/2013	juvenile idiopathic arthritis, Pn*	42081	1/16/2013	Negative	CMV10207	1/24/2013	HBoV2				C3453	2/1/2013	HBoV2
5	f	22.9m	7/5/2011	Nephrotic syndrome, bronchitis	31652	7/15/2011	HBoV1	h3572	7/4/2011	Negative						
6	m	11.5m	1/4/2013	septicemia, Pn*	42310	1/22/2013	Negative	CMV10428	1/24/2013	Negative				C3446	1/21/2013	HBoV2
7	f	25.5m	10/13/2011	lymphoblastic leukemia, Pn*	33658	11/29/2011	HBoV1	h3775	10/14/2011	Negative						
8	f	5.7m	6/28/2012	Langerhans cell hyperplasia, acute bronchitis	37746	7/5/2012	Negative	h4704	6/29/2012	Negative						
					38151	7/30/2012	HBoV1									

*HBoV, human bocavirus; Pn, pneumonia; CSF, cerebrospinal fluid; RV, rotavirus

Case 1, diagnosed with severe pneumonia and acute gastroenteritis, was assessed as HBoV1-positive in respiratory (49868; collected on 25 November, negative for respiratory virus screening), serum (CMV14456; collected on 25 November, negative for rotavirus), and fecal (CR9245, collected on 26 November, negative for rotavirus) specimens. Case 2, diagnosed with pneumonia and purulent meningitis, was also positive for HBoV1 in respiratory (48182; collected on 17 September) and serum (CMV13190; collected on 16 September) specimens.

Case 3, diagnosed with severe pneumonia, was negative for respiratory virus screening and positive for HBoV1 in a respiratory specimen (30789; collected on 12 May). However, acute diarrhea appeared 4 days later, nine times per day, and his fecal specimen (CR7110) was positive for both rotavirus and HBoV1.

Case 4, diagnosed with juvenile idiopathic arthritis and pneumonia with febrile convulsion, had HBoV2-positive CSF (C3453; collected on 1 February) and serum (CMV10207; collected on 24 January) specimens. However, her respiratory specimen (42081; collected on 16 January) was HBoV-negative.

Cases 5, 7, and 8 showed similar clinical courses to each other. These patients were admitted for non-infectious diseases (nephrotic syndrome, acute lymphoblastic leukemia, and Langerhans cell hyperplasia, respectively), and all developed respiratory infections 10 days later and were confirmed as HBoV1-positive.

Case 6, which developed infection after repair surgery for multiple intestinal fistulas and was diagnosed with septicemia and pneumonia with febrile convulsion, had an HBoV2-positive CSF specimen (C3446; collected on 21 January). However, his respiratory (42310; collected on 22 January) and serum (CMV10428; collected on 24 January) specimens were HBoV-negative.

## Discussion

Since its discovery in 2005, four main genotypes of HBoV have been identified globally [20, 21]. However, as Schildgen et al. [19] described, viral infections in general and those caused by HBoV in particular are more complicated than previously believed. We confidently believe clinical information obtained from medical records may provide more meaningful information to explain tissue tropism of different HBoVs, and our study of specimens from patients with various infectious diseases confirmed the necessity of obtaining all infectious disease data, including those reported previously [18].

In the present study, HBoV1 was the only genotype detected in patients with respiratory infections, while HBoV2 was predominantly detected in patients with gastrointestinal, hematological, and central nervous system infections. However, HBoV3 was rarely detected, and no HBoV4 strain was detected in this study.

Our phylogenetic analysis, based on more varied infectious diseases than those reported previously, indicated that HBoV1 sequences were highly conserved (99–100%) across the 8-year study period. Only a single genetic lineage of HBoV1 was circulating among children in China. However, 38 NP1/VP1 boundary nucleotide sequences of HBoV2 clustered into two distinct branches with substantial diversity, with variations up to 7.70%. We recently revealed that intra-genotype recombination occurred between HBoV2A and HBoV2B in children in China [18]. The present expanded study confirmed our finding that a new cluster of HBoV2 is prevalent in China.

No significant difference in the HBoV positivity rate between male and female patients or obvious seasonal distribution was observed in this study. Mitui et al. [22] reported an HBoV-positive rate in CSF specimens of 5.8%, which comprised two HBoV1 and two HBoV2 isolates. Additionally, Mori et al. [23] reported an HBoV-positive rate in CSF specimens of 3% (5/164), which comprised three HBoV1, one HBoV2, and one HBoV3 isolate. In the current study, the HBoV2-positive rate in CSF specimens was higher than that of HBoV1 (HBoV2: 0.76%, 17/2,239 vs. HBoV1: 0.04%, 1/2,239), suggesting a preference for HBoV2 in CSF specimens from Chinese patients. Moreover, some serum specimens were HBoV2-positive, contrary to the results of Tozer et al. [7], who reported that only HBoV1 DNA was found in serum specimens. Furthermore, Kantola et al. [24] reported that, overall, HBoV2 and possibly HBoV3 viremia appears to be rare. Therefore, further studies are needed to determine tissue tropism of different HBoVs.

In our case series, several clinical characteristics of HBoV-positive patients were identified. In Case 1, HBoV1 was identified in respiratory, serum, and fecal specimens collected on the same day, and in Case 2, both the respiratory and serum specimens were HBoV1-positive. These results may indicate that HBoV1 can cause not only respiratory infection, but also acute gastroenteritis and viremia. However, the diagnosis of HBoV infection based on PCR analysis alone is not adequate because of prolonged HBoV positivity in both respiratory and gastrointestinal tracts, especially at low copy numbers, leading to high detection rates in asymptomatic subjects. Thus, serology and/or HBoV1 DNA detection in the serum is essential to confirm acute HBoV1 infection-induced illness [2]. Additionally, cases 5, 7, and 8 developed respiratory infections after hospitalization for more than 10 days and were confirmed as HBoV1-positive, implying that HBoV1 can cause nosocomial infections. Case 3 was diagnosed with pneumonia, and respiratory specimens were confirmed as HBoV1-positive. Although the fecal specimen collected after the appearance of acute diarrhea was also HBoV1-positive, we believe that acute diarrhea was caused by rotavirus, and the patient had a nosocomial infection. Thus, the presence of HBoV1 in the fecal specimen may be the result of viral clearance following respiratory infection, which supports prolonged shedding of HBoV [2]. Lastly, Case 4, diagnosed with juvenile idiopathic arthritis and pneumonia, had HBoV2-positive CSF and serum specimens, suggesting that HBoV2 can infect the host through sites other than the respiratory tract or the respiratory tract is not susceptible to HBoV2. This was supported by previous findings of HBoV2 in stool and not airway samples [21].

In conclusion, our results are in line with earlier studies that HBoV1 was the predominant HBoV in respiratory samples and was also detectable in fecal, serum, and CSF specimens. Moreover, a single genetic lineage of HBoV1 was circulating among children in China during the study period, which may be a viral pathogen of respiratory infection. HBoV2 was the second most prevalent HBoV and commonly detected in stool specimens. Additionally, a new cluster of HBoV2, via intra-genotype recombination between HBoV2A and HBoV2B, was prevalent in China. The clinical data based on our case series provided some insight into the interpretation of molecular findings that HBoV1 can cause not only respiratory, but also gastroenteritis infection, as well as nosocomial infection, whereas HBoV2 can infect the host through sites other than the respiratory tract. Unfortunately, only several cases with different specimen types were available because of the retrospective nature of this study. Moreover, PCR alone is not an optimal diagnostic method of HBoV infection. Therefore, phylogenetic analysis and serological study of HBoV isolates from HBoV-positive patients with more varied specimen types available should be conducted in a prospective study.
